# Supplementary material for: Geo-Epidemiology of Malaria at the Health Area Level, Dire Health District, Mali, 2013–2017
Source: Int J Environ Res Public Health. 2020 Jun 4;17(11):3982. doi: 10.3390/ijerph17113982 (PMC7312793; doi:10.3390/ijerph17113982)
Supplement: Supplementary file 1 [file ijerph-17-03982-s001.pdf]

Supplementary Materials

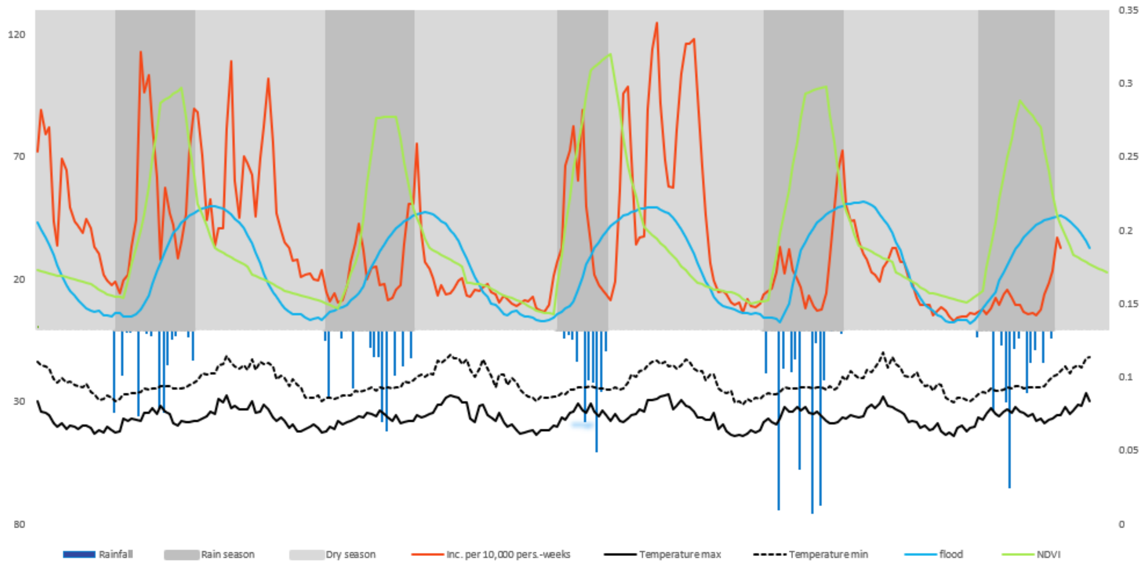

Figure S1. Evolution of weekly malaria incidence according to lagged meteorological factors in Dire health district, 2013–2017. The incidence per 10,000 person-weeks is represented by the red line, lagged NDVI by the light green line (On the secondary axis), and lagged river height (dm) by the light blue line. Lagged weekly rainfall accumulation (mm) is represented by the dark blue histogram, lagged median maximum temperature (°C) by the solid black line, and lagged median minimum temperature (°C) by the dashed black line. Dry seasons are represented by the light grey bar, and rainy seasons by the dark grey bar.

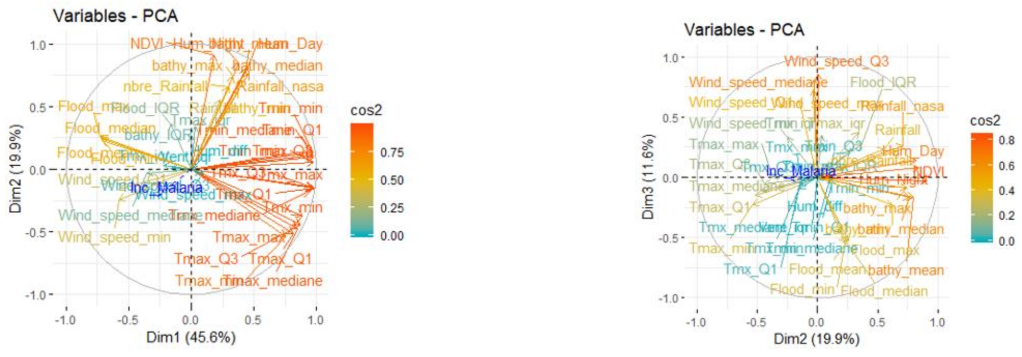

Figure S2. Graphic representation of the synthetic indicators generated by principal component analysis; SI 1 corresponds to temperature vs. river height; SI 2 corresponds to rainfall, humidity, bathymetry, and NDVI; SI 3 corresponds to wind speed.

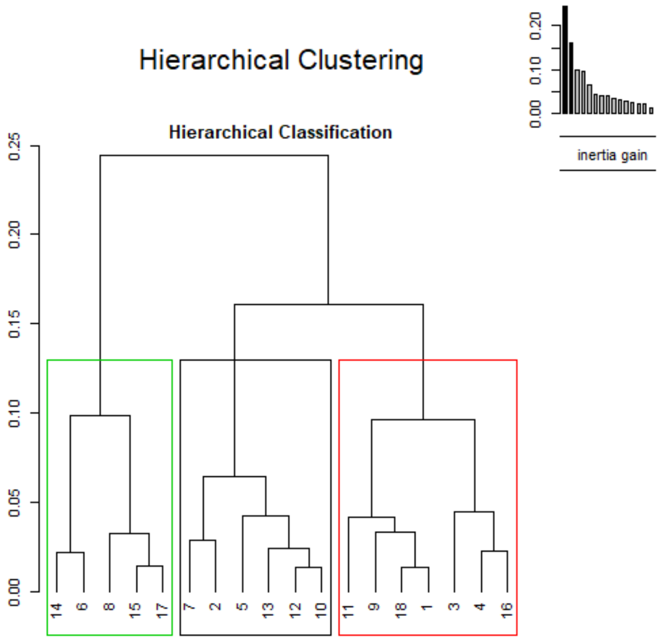

Figure S3. Hierarchical classification of land use variables showed 3 distinct risk classes with specific intra-class characteristics and inter-class differences.

Table S1. Malaria incidence by health area and time period.

ID	Health Area	Period 1	Period 2	Period 3	Period 4	Period 5	Period 6	Period 7	Period 8	Period 9
1	Alwalidji	85.10	110.84	58.80	27.29	89.24	75.60	38.08	36.08	13.49
2	Arham	85.86	75.05	23.23	35.10	40.06	107.50	17.80	22.87	7.68
3	BSA	31.82	49.94	23.07	16.76	21.85	69.63	9.10	28.03	3.92
4	Chirfiga	60.76	49.45	29.13	20.58	28.89	53.70	13.48	53.54	12.06
5	Dangha	82.63	68.55	23.39	35.12	48.89	82.76	23.29	29.04	19.60
6	Dire	33.71	3623	17.11	11.36	24.79	32.04	11.73	17.05	5.98
7	Gari	104.71	101.98	42.56	51.00	85.84	112.69	52.23	59.33	20.58
8	Haibongo	18.39	13.36	4.31	3.37	8.67	22.25	4.66	12.19	5.54
9	Kabaika	105.01	90.60	28.33	39.96	82.82	120.10	18.53	49.50	6.80
10	Kirchamba	65.55	59.28	32.16	23.22	43.18	57.39	20.13	25.28	18.01
11	Kondi	75.14	69.26	29.46	29.12	86.35	70.88	29.13	28.69	14.90
12	Koura	133.82	129.07	75.94	81.95	53.93	63.99	39.00	43.74	12.63
13	Garbacoira	54.77	42.93	15.55	7.83	39.66	74.42	19.32	24.10	6.00
14	Tienkour	39.87	57.12	11.59	8.32	60.78	73.10	15.75	25.52	3.53
15	Tindirma	61.28	54.45	26.29	33.48	44.12	57.14	24.52	26.66	4.28
16	Salakoira	74.01	51.27	34.22	20.64	90.43	79.78	53.50	47.21	17.39
17	Sarayamou	23.46	13.88	8.12	4.38	19.15	23.26	7.34	8.69	3.32
18	Issafaye	179.79	95.15	38.58	51.68	72.49	122.98	31.74	56.46	8.56

Maximum malaria incidence per time period decreased from 179.79 to 20.58 cases per 10,000 person-weeks over the study period.

Table S2. Meteorological and environmental variables and their source.

Variables collected at the health district level.		
**Variables**	**Resolution**	**Source**
Rainfall		Local agricultural service
Relative humidity	1°	AIRS
Wind Speed	0.25°	GLDAS
River height		Local agricultural service
Temperature	0.5 x 0.625°	MERRA-2
NDVI	0.05°	MODIS Terra
**Variables collected at the health area level**		
Rainfall	0.1°	IMERG V6
Off-season cultivation		Local agricultural service
Cultivated land area		Local agricultural service
Off-season cultivation		Field study
Presence of lowlands		Field study
Proximity to the river		Field study
Propensity for flooding		Field study
Population		National data

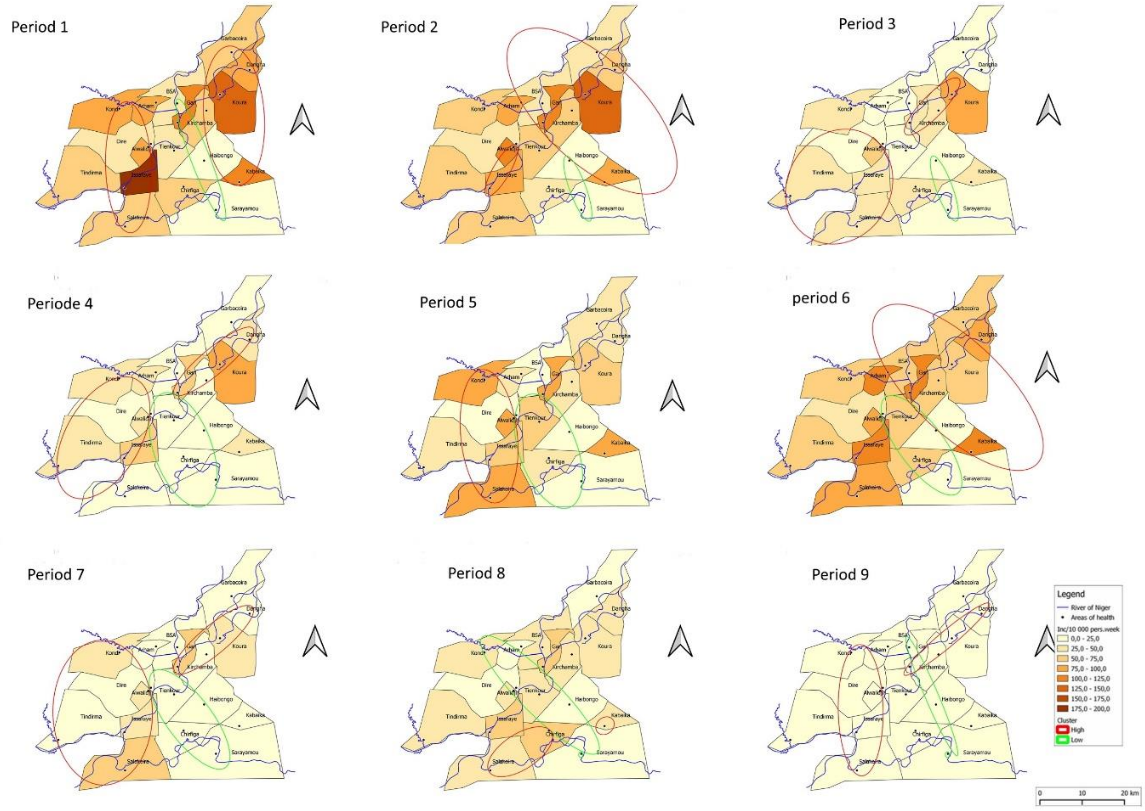

Figure S4. Maps of malaria incidence and hotspot/coldspots by time period for the 18 health areas. Malaria incidence declined gradually over time, but the risk of a health area being a hotspot was almost permanent in the north and west; the central, populated urban area was protected during all time periods.
